# Efficacy and safety of reperfusion treatments in middle-old and oldest-old stroke patients

**DOI:** 10.1007/s10072-022-05958-4

**Published:** 2022-02-24

**Authors:** Giovanna Viticchi, Eleonora Potente, Lorenzo Falsetti, Marco Burattini, Marco Bartolini, Laura Buratti, Giuseppe Pelliccioni, Mauro Silvestrini

**Affiliations:** 1 Neurological Clinic, Marche Polytechnic University, Via Conca 1, 60020 Ancona, Italy; 2 Neurology Department, IRCCS INRCA, Ancona, Italy; 3 Internal and Subintensive Medicine, Ospedali Riuniti, Ancona, Italy

**Keywords:** Middle-old, Oldest-old, Stroke, Thrombolysis, Thrombectomy

## Abstract

**Introduction:**

Intravenous thrombolysis (IT) and mechanical thrombectomy (MT) have significantly changed the clinical outcome of acute ischaemic stroke (AIS). Concerns about possible complications often reduce the use of these treatment options for older patients, preferentially managed with antiplatelet therapy (AT). Aim of this study was to evaluate, in a population of middle-old (75–84 years) and oldest-old (≥ 85 years) subjects, the efficacy and safety of different treatments for AIS (IT, IT + MT, MT or AT), mortality and incidence of serious complications.

**Patients and methods:**

All patients aged over 75 years admitted for AIS in two Stroke Units were enrolled. The physician in each case considered all treatment options and chose the best approach. NIHSS and modified Rankin Scale (mRS) were obtained and differences between admission and discharge scores, defined as delta(NIHSS) and delta(mRS), were calculated. The relationship between delta(NIHSS), delta(mRS) and type of procedure was analysed with a GLM/Multivariate model. Differences in mortality and incidence of serious complications were analysed with the chi-square test.

**Results:**

A total of 273 patients, mean age 84.07 (± 5.47) years, were included. The Delta(NIHSS) was significantly lower in patients treated with AT than in those treated with IT and MT (*p* < 0.009 and *p* < 0.005, respectively). Haemorrhagic infarction occurrence was significantly lower (*p* < 0.0001) among patients treated with AT (10.6%) or IT (16.7%) compared to MT (34.9%) or MT + IT (37.0%). No significant difference was observed for in-hospital mortality. Age did not influence the outcome.

**Conclusions:**

Our results suggest that IT and AT are effective and relatively safe approaches in middle-aged and older patients.

## Introduction

Intravenous thrombolysis (IT) and mechanical thrombectomy (MT) are the most effective treatments for acute ischaemic stroke (AIS). Recently, due to the results of international studies, the time window for these treatments has been extended to 9 h after symptom onset for IT and to 24 h for MV [1, 2]. Furthermore, guidelines do not set limits based on the age of patients [3]. However, in clinical practice, older patients are often treated less aggressively than younger subjects [4], probably because they may have a higher risk of complications, including brain haemorrhage, infection or death. Several studies confirm that older age is an independent negative predictor of AIS outcomes [5]. In addition, the vast majority of trials exploring the efficacy and safety of reperfusion therapies have not included subjects ≥ 80 years of age, with the notable exception of the third international stroke trial (IST3) [6], reducing information for proper therapeutic management of older patients. On the other hand, improvements in therapeutic approaches and early diagnosis, innovation in resuscitation techniques, and increasing attention to healthy lifestyles have increased the average life span in all industrialised countries.

The aim of this study was to explore the characteristics and severity of AIS in a cohort of elderly subjects, comparing middle-old (75–84 years) and oldest-old (85 years) subjects, according to recent classifications [7]. We evaluated the efficacy of different AIS treatments: IT, IT + MT, MT or antiplatelets (AT) in the hyperacute phase and the safety of these therapies by assessing mortality and risk of haemorrhagic infarction (HI).

## Patients and methods

This study was performed in two Stroke Units (Azienda Ospedaliero-Universitaria di Ancona; Istituto di Ricerca Geriatrica INRCA, Ancona), enrolling patients aged at least 75 years, who were admitted to the Emergency Department for AIS over a 4-year period (01/01/2017–31/12/2020).

For each patient, we collected a complete medical history. A clinical evaluation and cerebral Angio-CT was also performed. In selected cases, such as in waking strokes, we performed brain perfusion CT or brain MRI. If patients were unable to provide reliable information, we contacted their caregivers.

Inclusion criteria were as follows: (a) AIS; (b) age ≥ 75 years; Exclusion criteria were as follows: (a) absent or incomplete clinical data; (b) brain haemorrhage at the first brain scan; (c) brain tumours, cerebral arteriovenous malformations or other brain lesions that could potentially act as confounders.

Each patient was referred to IT, IT + MT, MT or AT according to international guidelines. For each patient, the physician considered all therapeutic options without any limitation and, finally, based on the indications according to the guidelines or for the presence of ineligibility criteria, based on clinical, laboratory or radiological examinations, indicated the best therapeutic approach and discarded the impracticable options. We performed a follow-up brain scan 24 h after treatment: in the absence of bleeding, we started or continued AT. Each subject was clinically assessed with the National Institutes of Health Stroke Scale (NIHSS) and the modified Rankin Scale (mRS) at admission and discharge. The NIHSS and mRS are the most validated scores in the literature for assessing severity and dependence after stroke, respectively. Specifically, the mRS is a score to evaluate the degree of disability or dependence in activities of daily living of people who have suffered a stroke or other neurological causes of disability. It is usually used to quantify the degree of autonomy at home, but we chose to use it because during hospitalisation each patient was assessed daily by a physiotherapist to assess the degree of autonomy after the stroke, and the mRS score at discharge expresses this assessment.

The collection of the clinical history focused on comorbidities (new-onset or pre-existing atrial fibrillation (AF), coronary artery disease, OSAS, previous AIS, systemic hypertension, diabetes, dyslipidaemia and smoking). We performed neck vessel assessment and transcranial Doppler and, according to the clinical and instrumental findings, our patients were classified according to the Bamford classification: total anterior circulation stroke (TACS), partial anterior circulation stroke (PACS), lacunar stroke (LACS) and posterior circulation stroke (POCS) [8].

### Compliance with ethical standards

The authors declare that they had no conflicts of interest. The Ethics Committee of the Marche Region (CERM), Italy, approved the study. All participants and/or caregivers gave written informed consent to participate and were treated according to the 1964 Declaration of Helsinki and its subsequent amendments or comparable ethical standards.

### Statistical analysis

We adopted the analytical strategies for NIHSS and mRS suggested by the ESO in 2012 [9] and considered NIHSS, mRS and their difference from discharge to admission (delta) as continuous variables. We chose to adopt delta(mRS) and delta(NIHSS) as outcome measures because delta is less dependent on NIHSS and/or mRS at baseline and allows us to provide a more easily interpretable outcome measure for each procedure [9]. Age, admission and discharge NIHSS, and admission and discharge mRS were collected as continuous variables. We calculated (i) the difference between admission and discharge NIHSS, delta(NIHSS) and (ii) the difference between admission and discharge mRS, delta(mRS). Sex, comorbidity, significant carotid stenosis (CS) ipsilateral or contralateral to the side of the stroke, use of anticoagulant or antiplatelet drugs before admission and the occurrence of HI after the procedure were treated as dichotomous values. Marital status, smoking, type of stroke, type of procedure, clinical outcome and discharge treatment were summarised as categorical variables.

Continuous variables were tested for normality with the Komologorov-Smirnov test: normally distributed variables were presented as mean and standard deviation (SD) and compared with *t*-test. Non-normally distributed variables were presented as median and interquartile range [IQR] and compared with Mann–Whitney *U*-test. Dichotomous and categorical variables were compared with chi-squared test. In order to assess the different distribution of delta(NIHSS) and delta(mRS) among different procedures, we graphed the differential distributions of these variables and compared them with the non-parametric Kruskal–Wallis test.

The association among variables was evaluated with Pearson’s bivariate test: variables significantly associated with delta(NIHSS) or delta(mRS) were selected for multivariate analysis. Our GLM/Multivariate model considered (i) delta(NIHSS) and delta(mRS) as dependent variables; (ii) procedure type, stroke site, HI and CS ipsilateral to stroke as independent variables; and (iii) age and sex as covariates. Statistical analysis was performed with SPSS 13.0 for Windows Systems.

## Results

Of 368 patients, 95 were excluded: 57 for intracranial haemorrage, 22 for potentially confounding brain lesions, 16 for incomplete clinical data or caregiver absence, obtaining a final cohort of 273 patients. Baseline characteristics, subdividing the cohort into middle-old (75–84 years; *n* = 161) and oldest-old patients (≥ 85 years; *n* = 112), are synthesised in Table 1.

**Table 1:** Baseline characteristics of the sample

	Overall (*n* = 273)	Age 75–84 (*n* = 161)	Age ≥ 85 (*n* = 112)	*p*
*General information*				
Age (mean ± SD), years	84.07 (± 5.46)	80.61 (± 3.09)	89.86 (± 3.17)	0.0001
Age ≥ 85 years (*n*, %)	112 (41.0%)	–	–	–
Female sex (*n*, %)	169 (61.9%)	100 (58.5%)	69 (67.6%)	0.131
Marital status				
Married (*n*, %) Non-married (*n*, %) Widow (*n*, %)	120 (44.0%) 53 (19.4%) 100 (36.6%)	32 (11.7%) 80 (29.3%) 59 (21.6%)	21 (7.7%) 40 (14.7%) 41 (15.0%)	0.469
*Vascular risk factors*				
Diabetes (*n*, %)	71 (26.0%)	45 (16.5%)	26 (9.5%)	0.880
Hypertension (*n*, %)	202 (74.0%)	128 (46.9%)	74 (27.1%)	0.675
Dyslipidaemia (*n*, %)	118 (43.2%)	80 (29.3%)	38 (13.9%)	0.124
Smoking status				
Non-smoker (*n*, %) Active smoker (*n*, %) Previous smoker (*n*, %)	174 (63.7%) 24 (8.8%) 75 (27.5%)	104 (38.1%) 17 (6.2%) 50 (18.3%)	70 (25.6%) 7 (2.6%) 25 (9.2%)	0.402
Previous stroke (*n*, %)	58 (21.2%)	34 (12.5%)	24 (8.8%)	0.476
Previous ACS (*n*, %)	43 (15.8%)	26 (9.5%)	17 (6.2%)	0.748
OSAS (*n*, %)	3 (1.1%)	1 (0.4%)	2 (0.7%)	0.291
Pre-existing NVAF (*n*, %)	82 (30.0%)	46 (16.8%)	36 (13.2%)	0.143
New-onset NVAF (*n*, %)	63 (23.1%)	40 (14.7%)	23 (8.4%)	0.873
Carotid stenosis, ipsilateral (*n*, %)	24 (8.8%)	15 (5.5%)	9 (3.3%)	0.988
Carotid stenosis, contralateral (*n*, %)	19 (7.0%)	10 (3.7%)	9 (3.3%)	0.350
Antiplatelet drugs (*n*, %)	114 (41.8%)	72 (26.4%)	42 (15.4%)	0.418
Anticoagulant drugs (*n*, %)	55 (20.1%)	29 (10.6%)	26 (9.5%)	0.089
*Stroke type and procedures*				
NIHSS at admission (median, [IQR])	9 [13]	10 [13]	8 [12]	0.743
NIHSS at discharge (median, [IQR])	4 [12]	4 [11]	4 [12]	0.747
Delta(NIHSS) (median, [IQR])	2 [5]	2.50 [5]	2 [4]	0.300
mRS at admission (median, [IQR])	0 [2]	0 [2]	2 [3]	0.0001
mRS at discharge (median, [IQR])	4 [3]	3 [4]	4 [2]	0.016
Delta(mRS) (median, [IQR])	− 2 [4]	− 2 [3]	− 1 [3]	0.140
Stroke type				
TACI (*n*, %) LACI (*n*, %) PACI (*n*, %) POCI (*n*, %)	10 (40.3%) 46 (16.8%) 73 (26.7%) 44 (16.1%)	70 (25.6%) 42 (15.4%) 32 (11.7%) 27 (9.9%)	40 (14.7%) 31 (11.4%) 14 (5.1%) 17 (6.2%)	0.608
Procedure type				
Antiplatelet therapy (*n*, %) Fibrinolysis (*n*, %) Thrombectomy (*n*, %) Fibrinolysis, thrombectomy (*n*, %)	94 (34.4%) 90 (33.0%) 43 (15.8%) 46 (16.8%)	51 (18.7%) 49 (17.9%) 30 (11.0%) 41 (15.0%)	43 (15.8%) 41 (15.0%) 13 (4.80%) 5 (1.8%)	0.0001
Haemorragic infarction (*n*, %)	57 (20.9%)	34 (12.5%)	23 (22.5%)	0.600
*Outcome and treatments*				
Discharge				
Discharged at home (*n*, %) Transfer to another hospital (*n*, %) Post-acute department (*n*, %) Long-term care facilities (*n*, %) Neurorehabilitation (*n*, %) ICU (*n*, %) Death (*n*, %)	103 (37.7%) 65 (23.8%) 25 (9.1%) 9 (3.3%) 39 (14.3%) 12 (4.4%) 20 (7.3%)	67 (24.5%) 48 (17.6%) 10 (3.7%) 4 (1.5%) 24 (8.8%) 7 (2.6%) 11 (4.0%)	36 (13.2%) 17 (6.2%) 15 (5.5%) 5 (1.8%) 15 (5.5%) 5 (1.8%) 9 (3.3%)	0.130
Discharge treatment				
Aspirin (*n*, %) Clopidogrel (*n*, %) Aspirin and clopidogrel (*n*, %) DOACs (*n*, %) LMWH (*n*, %) Ticlopidine (*n*, %) Warfarin (*n*, %) No therapy (*n*, %)	120 (44.0%) 34 (12.5%) 10 (3.7%) 46 (16.8%) 18 (6.6%) 2 (0.7%) 8 (2.9%) 35 (12.8%)	77 (28.2%) 26 (9.5%) 9 (3.3%) 21 (7.7%) 12 (4.4%) 1 (0.4%) 6 (2.2%) 19 (7.0%)	43 (15.8%) 8 (2.9%) 1 (0.4%) 25 (9.2%) 6 (2.2%) 1 (0.4%) 2 (0.7%) 16 (5.9%)	0.059

**Legend:** *ACS*, acute coronary syndrome; *DOACs*, direct oral anticoagulants; *ICU*, intensive care unit; *LACI*, lacunar cerebral infarction; *LACI*, lacunar cerebral infarction; *LMWH*, low molecular weight heparin; *mRS*, modified Rankin scale; *NIHSS*, National Institutes of Health Stroke Scale; *NVAF*, non-valvular atrial fibrillation; *PACI*, partial anterior cerebral infarction; *POCI*, posterior cerebral infarction; *SD*, standard deviation; *TACI*, total anterior cerebral infarction

We observed a significant difference between procedures, as shown in Tables 2 and 3: subjects undergoing MT + IT had worse admission(NIHSS), discharge(NIHSS), admission(mRS) and discharge(mRS) with respect to patients treated with IT or AT, while these scores were similar between MT + IT and MT.

**Table 2:** Mean of pre-procedure NIHSS, discharge NIHSS, Delta(NIHSS) and pre-procedure mRS, discharge mRS, Delta(mRS) according to procedure type

Dependent variable	Procedure	*N*	Mean	95%CI for mean	
				Lower bound	Upper bound
Admission NIHSS	Fibrinolysis	90	9.54	8.2	10.88
	Thrombectomy	43	15.09	13.56	16.61
	Fibrinolysis + thrombectomy	46	16.45	15.29	17.61
	Antiplatelet	94	5.71	4.64	6.77
	Total	273	10.26	9.45	11.07
Discharge NIHSS	Fibrinolysis	90	5.72	4.22	7.22
	Thrombectomy	43	10.41	8.38	12.44
	Fibrinolysis + thrombectomy	46	12.52	10.44	14.6
	Antiplatelet	93	4.69	3.42	5.97
	Total	272	7.26	6.38	8.14
Delta(NIHSS)	Fibrinolysis	90	3.82	2.51	5.13
	Thrombectomy	43	4.67	2.78	6.56
	Fibrinolysis + thrombectomy	46	3.93	1.88	5.98
	Antiplatelet	94	1.06	0.33	1.78
	Total	273	3.02	2.34	3.7
Admission mRS	Fibrinolysis	90	1.32	1	1.64
	Thrombectomy	43	0.37	0.13	0.6
	Fibrinolysis + thrombectomy	46	0.45	0.17	0.74
	Antiplatelet	94	1.82	1.51	2.14
	Total	273	1.2	1.02	1.37
Discharge mRS	Fibrinolysis	90	2.93	2.52	3.34
	Thrombectomy	43	3.79	3.36	4.21
	Fibrinolysis + thrombectomy	46	4.13	3.71	4.54
	Antiplatelet	94	2.85	2.46	3.23
	Total	273	3.24	3.02	3.46
Delta(mRS)	Fibrinolysis	90	− 1.61	− 1.97	− 1.24
	Thrombectomy	43	− 3.41	− 3.84	− 2.99
	Fibrinolysis + thrombectomy	46	− 3.67	− 4.15	− 3.18
	Antiplatelet	94	− 1.02	− 1.29	− 0.75
	Total	273	− 2.04	− 2.26	− 1.81

**Legend:** *CI*, confidence interval; *mRS*, modified Rankin scale; *NIHSS*, National Institutes of Health Stroke Scale

**Table 3:** Comparisons of pre-procedure NIHSS, discharge NIHSS, Delta(NIHSS) and pre-procedure mRS, discharge mRS, Delta(mRS) according to procedure type

Dependent variable	Procedure		(I–J)	*p*	95%CI	
	(I)	(J)			Lower	Upper
Admission NIHSS	Fibrinolysis	Thrombectomy	− 5.54	0.0001	− 7.52	− 3.57
		Fibrinolysis + thrombectomy	− 6.91	0.0001	− 8.84	− 4.98
		Antiplatelet	3.83	0.0001	2.26	5.4
	Thrombectomy	Fibrinolysis	5.54	0.0001	3.57	7.52
		Fibrinolysis + thrombectomy	− 1.36	0.235	− 3.62	0.89
		Antiplatelet	9.38	0.0001	7.42	11.34
	Fibrinolysis + thrombectomy	Fibrinolysis	6.91	0.0001	4.98	8.84
		Thrombectomy	1.36	0.235	− 0.89	3.62
		Antiplatelet	10.74	0.0001	8.82	12.65
	Antiplatelet	Fibrinolysis	− 3.83	0.0001	− 5.4	− 2.26
		Thrombectomy	− 9.38	0.0001	− 11.34	− 7.42
		Fibrinolysis + thrombectomy	− 10.74	0.0001	− 12.65	− 8.82
Discharge NIHSS	Fibrinolysis	Thrombectomy	− 4.69	0.0002	− 7.14	− 2.24
		Fibrinolysis + thrombectomy	− 6.79	0.0001	− 9.19	− 4.4
		Antiplatelet	1.02	0.303	− 0.93	2.97
	Thrombectomy	Fibrinolysis	4.69	0.0002	2.24	7.14
		Fibrinolysis + thrombectomy	− 2.1	0.141	− 4.9	0.7
		Antiplatelet	5.71	0.0001	3.28	8.15
	Fibrinolysis + thrombectomy	Fibrinolysis	6.79	0.0001	4.4	9.19
		Thrombectomy	2.1	0.141	− 0.7	4.9
		Antiplatelet	7.82	0.0001	5.43	10.2
	Antiplatelet	Fibrinolysis	− 1.02	0.303	− 2.97	0.93
		Thrombectomy	− 5.71	0.0001	− 8.15	− 3.28
		Fibrinolysis + thrombectomy	− 7.82	0.0001	− 10.2	− 5.43
Delta(NIHSS)	Fibrinolysis	Thrombectomy	− 0.85	0.411	− 2.89	1.18
		Fibrinolysis + thrombectomy	− 0.11	0.911	− 2.1	1.88
		Antiplatelet	2.75	0.009	1.13	4.37
	Thrombectomy	Fibrinolysis	0.85	0.411	− 1.18	2.89
		Fibrinolysis + thrombectomy	0.73	0.532	− 1.59	3.07
		Antiplatelet	3.61	0.005	1.58	5.63
	Fibrinolysis + thrombectomy	Fibrinolysis	0.11	0.911	− 1.88	2.1
		Thrombectomy	− 0.73	0.532	− 3.07	1.59
		Antiplatelet	2.87	0.005	0.89	4.84
	Antiplatelet	Fibrinolysis	− 2.75	0.009	− 4.37	− 1.13
		Thrombectomy	− 3.61	0.005	− 5.63	− 1.58
		Fibrinolysis + thrombectomy	− 2.87	0.005	− 4.84	− 0.89
Admission mRS	Fibrinolysis	Thrombectomy	0.95	0.0002	0.45	1.44
		Fibrinolysis + thrombectomy	0.86	0.0005	0.38	1.34
		Antiplatelet	− 0.5	0.0112	− 0.9	− 0.11
	Thrombectomy	Fibrinolysis	− 0.95	0.0002	− 1.44	− 0.45
		Fibrinolysis + thrombectomy	− 0.08	0.769	− 0.65	0.48
		Antiplatelet	− 1.45	0.0001	− 1.94	− 0.96
	Fibrinolysis + thrombectomy	Fibrinolysis	− 0.86	0.0005	− 1.34	− 0.38
		Thrombectomy	0.08	0.769	− 0.48	0.65
		Antiplatelet	− 1.37	0.0001	− 1.85	− 0.89
	Antiplatelet	Fibrinolysis	0.5	0.012	0.11	0.9
		Thrombectomy	1.45	0.0001	0.96	1.94
		Fibrinolysis + thrombectomy	1.37	0.0001	0.89	1.85
Discharge mRS	Fibrinolysis	Thrombectomy	− 0.85	0.009	− 1.5	− 0.21
		Fibrinolysis + thrombectomy	− 1.19	0.0002	− 1.82	− 0.56
		Antiplatelet	0.08	0.753	− 0.43	0.59
	Thrombectomy	Fibrinolysis	0.85	0.009	0.21	1.5
		Fibrinolysis + thrombectomy	− 0.33	0.366	− 1.07	0.39
		Antiplatelet	0.93	0.004	0.29	1.58
	Fibrinolysis + thrombectomy	Fibrinolysis	1.19	0.0002	0.56	1.82
		Thrombectomy	0.33	0.366	− 0.39	1.07
		Antiplatelet	1.27	0.0001	0.65	1.9
	Antiplatelet	Fibrinolysis	− 0.08	0.753	− 0.59	0.43
		Thrombectomy	− 0.93	0.004	− 1.58	− 0.29
		Fibrinolysis + thrombectomy	− 1.27	0.0001	− 1.9	− 0.65
Delta(mRS)	Fibrinolysis	Thrombectomy	1.8	0.0001	1.24	2.36
		Fibrinolysis + thrombectomy	2.06	0.0001	1.51	2.61
		Antiplatelet	− 0.58	0.009	− 1.03	− 0.14
	Thrombectomy	Fibrinolysis	− 1.8	0.0001	− 2.36	− 1.24
		Fibrinolysis + thrombectomy	0.25	0.433	− 0.38	0.89
		Antiplatelet	− 2.39	0.0001	− 2.95	− 1.84
	Fibrinolysis + thrombectomy	Fibrinolysis	− 2.06	0.0001	− 2.61	− 1.51
		Thrombectomy	− 0.25	0.433	− 0.89	0.38
		Antiplatelet	− 2.65	0.0001	− 3.19	− 2.1
	Antiplatelet	Fibrinolysis	0.58	0.009	0.14	1.03
		Thrombectomy	2.39	0.0001	1.84	2.95
		Fibrinolysis + thrombectomy	2.65	0.0001	2.1	3.19

**Legend:** *CI*, confidence interval; *mRS*, modified Rankin scale; *NIHSS*, National Institutes of Health Stroke Scale

**Fig. 1:**
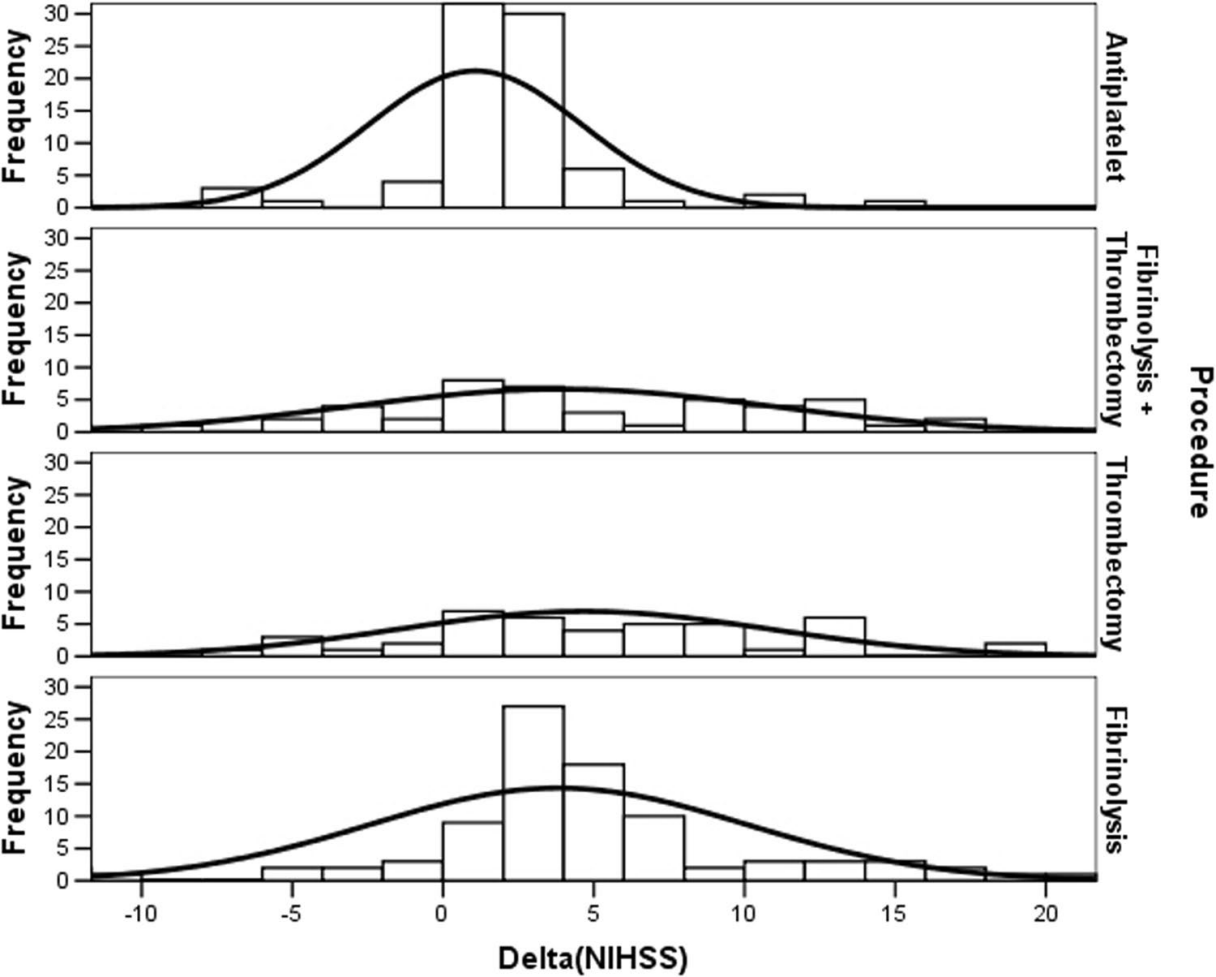
Different distribution of delta(NIHSS)

**Fig. 2:**
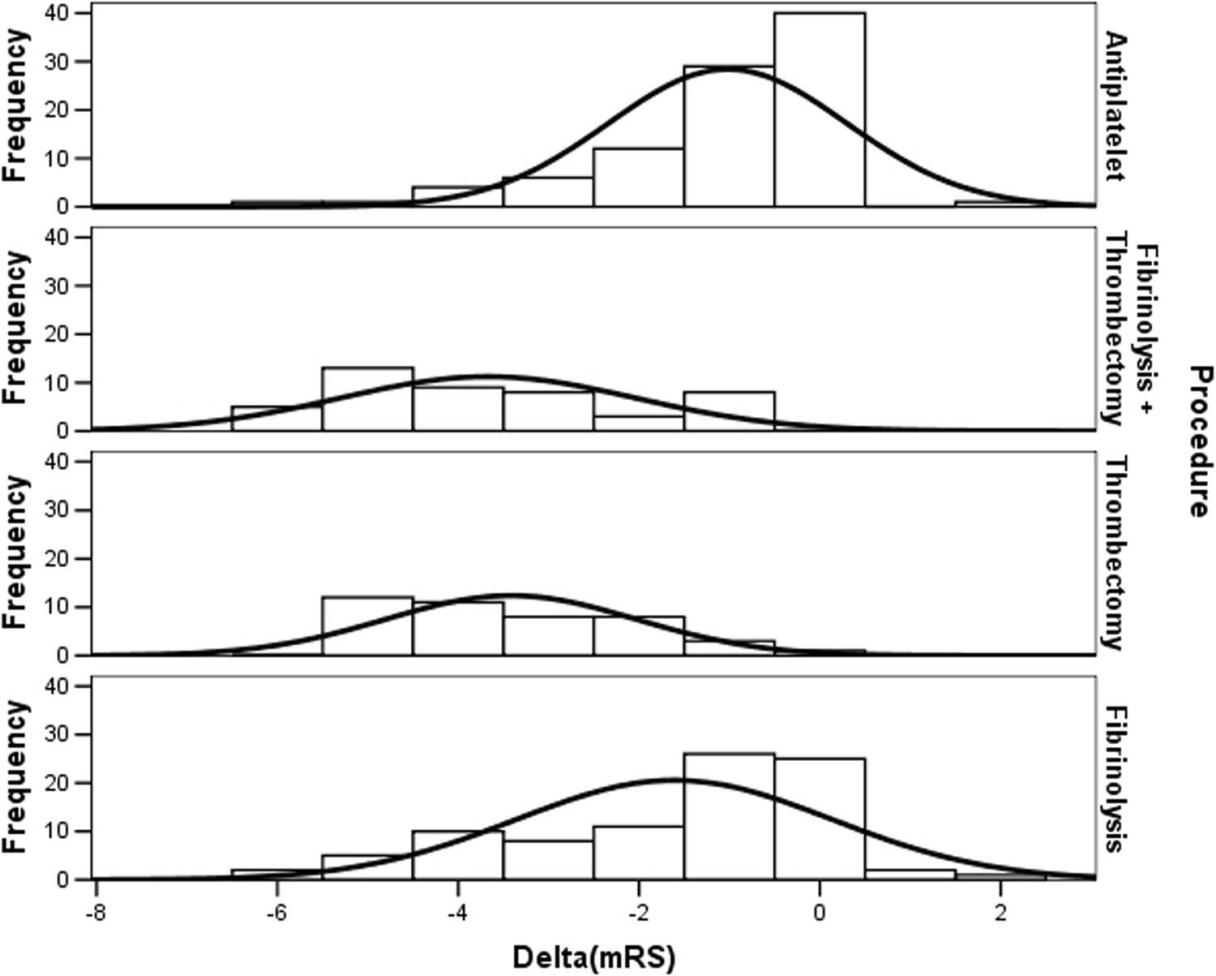
Different distribution of delta(mRS)

Delta(NIHSS) resulted significantly lower in AT, while other treatments were associated to similar scores (Tables 2 and 3). Delta(mRS) was similar between MT and MT + AT, while AT or IT were associated to lower scores (Tables 2 and 3). We graphed the different distribution of delta(NIHSS) in Fig. 1, underlining a significant delta(NIHSS) difference in the median distribution among different procedures (*p* = 0,0001; Kruskal–Wallis test); similarly, we graphed the different distribution of delta(mRS) in Fig. 2, underlining a significant delta(mRS) difference in the median distribution among different procedures (*p* = 0,0001; Kruskal–Wallis test).

Pearson’s bivariate test underlined that procedure type, stroke site, HI and CS ipsilateral to stroke were significantly associated to delta(NIHSS) and delta(mRS). GLM/Multivariate model resulted significant (*p* < 0.0001): we estimated the marginal means of delta(NIHSS) and delta(mRS) according to the performed procedure (Table 3).

Post-procedure HI was associated to a significant difference both in delta(NIHSS) (no HI: 3.023; 95%CI: 1.678–4.369; HI: 0.284; 95%CI: − 1.536–2.103; *p* = 0.002) and in delta(mRS) (no HI: − 2.357; 95%CI: (− 2.724)–(− 1.990); HI: − 3.228; 95%CI: (− 3.725)–(− 2.732); *p* = 0.0001).

Crosstabulating the procedure type with HI occurrence, we observed HI absence in a significantly higher (*p* < 0.0001) proportion of subjects treated with AT (30.8%) or IT (27.5%) when compared with MT (10.3%) and MT + IT (10.6%). HI was significantly lower (*p* < 0.0001) in AT (10.6%) or IT (16.7%) than MT (34.9%) or MT + IT (37.0%).

Crosstabulating the procedure type with in-hospital death, we observed that 8 subjects (2.9%) in the IT group, 6 (2.2%) in the MT + IT group, 6 (2.2%) in the AT group and no patients in the MT group died before discharge: these differences were not statistically significant (*p* = 0.109).

Stroke type was significantly (*p* = 0.014) associated to HI, which was more common in TACS (12.1%) than PACS (4.8%), LACS (2.6%) and POCS (1.5%). Comparing all the results between middle-old and oldest-old, we did not find any significant difference (Table 1).

## Discussion

Our results, obtained in a cohort of consecutive elderly subjects undergoing AIS treatment, showed that MT was used in patients with a more compromised neurological status. Patients who received IT and MT presented worse NIHSS and mRS scores at discharge but, considering the delta(NIHSS) and delta(mRS), these procedures, alone or in combination, provided the best neurological and functional outcome. IT presented a significantly better delta(NIHSS) than AT, but worse scores than MT. This is relevant because IT showed a low HI rate, similar to AT. Based on these data, IT seems to represent an accessible and relatively safe approach for AIS in the elderly, easier to implement than MT and more effective than AT.

Literature data express a favourable indication for IT in the elderly, although not all studies and trial results agree on its safety. The Endostroke Study found an indication for IT in elderly patients (≥ 80 years) with a prevalence of HI similar to that observed in the control group, but a higher mortality [10]. Other large studies have found similar results [11]. IST-3 showed greater improvement in patients older than 80 years with a similar 6-month mortality rate between young and old [6]. Recanalisation rates appeared to be similar in younger and older patients [11, 12]. Several other studies emphasised a worse outcome in patients aged ≥ 80 years after IT, such as in the CASES study [13].

These controversial results are probably due to the co-morbidities affecting elderly subjects and the high rate of complications during hospitalisation (cardiac, pulmonary and renal disorders, infections or prolonged bed rest) [13, 14].

In our study, the MT procedure, alone or associated with IT, presented the best results, with the highest delta(NIHSS) and delta(mRS). Data from the studies considered for approval of MT in clinical practice have shown that thrombectomy is also effective in populations ≥ 80 years old [15]. However, it is important to note that all these studies together enrolled only 198 patients ≥ 80 years. Almost all studies on this topic have found that older people undergoing MT have a worse outcome and higher mortality than younger people [16, 17].

Comparing MT and AT, ‘real-world’ experience has shown that patients referred to MT did not achieve a significant improvement in mRS and had a higher probability of HI [5]. On the other hand, some studies have found that MT, despite increased rates of brain haemorrhage and death, leads to better outcomes than non-invasive treatment [16, 18]. Our data showed that mortality does not discriminate significantly between the different treatments, confirming a higher prevalence of HI in subjects undergoing MT or MT + IT. A large meta-analysis observed a lower rate of successful recanalisation but not a significantly higher rate of symptomatic HI among the elderly [19]. Other reports pointed to higher rates of successful recanalisation, with a peak of 96% in patients aged 80 years [16, 20].

These findings can be variously interpreted. Older people present several features that could increase the risk of complications, such as cognitive impairment [21, 22], increased fragility of cerebral vessels with reduced extent of penumbra [18], increased extension of leukoaraiosis [19], amyloid angiopathy [23] and impaired neurological reserve [24]. The variable presence of all these elements could influence the different clinical consequences due to AIS. Furthermore, the results of interventional approaches might vary depending on the age of the patients or the anatomical state of the cerebral vessels. In the elderly, navigation of the device is often difficult and may require a change in the approach pathway, thus increasing MT time, duration of anaesthesia and the likelihood of post-procedural bleeding [20]. The Endostroke registry showed a progressively lower probability of achieving a small final infarct volume with increasing age and a higher rate — in older people — of so-called futile recanalisations. These aspects support the concept that age itself might be an independent factor for MT failure [10].

All the issues discussed could partly explain the worse results observed in elderly people undergoing IT and MT. Impaired macrocirculation and microcirculation could be associated in elderly subjects with a reduced adaptive response to ischemia, thus explaining reduced efficacy of these treatments in old age [23].

The second interesting feature of our study is that we found no significant difference between the population of middle-old and oldest-old subjects in all the parameters evaluated. The Copenhagen Stroke study found poor outcomes and high mortality in the very old, both in the short and long term, compared to younger patients. The authors stated that the complications and increased mortality were probably due to the large number of comorbidities, especially AF and previous disability [25, 26].

In our study, we directly compared middle-old to oldest-old and observed no significant differences between these two populations. The benefits due to IT and MT were also maintained in patients aged ≥ 85 years. Furthermore, we observed no significant difference between the two populations for HI and death. According to some studies, older patients seemed to be at higher risk for complications and death, mainly because of pre-stroke conditions and comorbidities, not because of age per se [25]. These findings are only apparently at odds with the well-established fact that older age is an independent risk factor for poor post-stroke prognosis. On the contrary, our observation underlines the need for a better selection of patients for IT or MT, because elderly people with multiple comorbidities seem to be at higher risk of complications.

The main limitation of this study is due to its retrospective nature. Furthermore, due to the small number of patients in each group, we could not differentiate between different types of stroke or other factors including type of anaesthesia or time spent in the Stroke Unit. The utilisation of the main outcome measures (mRS, NIHSS and their delta between admission and discharge) as continuous variables, albeit commonly performed in several studies and allowed by guidelines, could be difficult to interpret, since the original dimensionality of these variables is categorical; however, with these analyses, we were able to underline even small differences that could be useful for common practice.

In addition, we did not use a randomised procedure to allocate each patient to a different treatment group, but in each case, the physician evaluated all available treatment options for the patient and chose the best applicable one. This was done according to the international guidelines for stroke, which clearly indicate to consider all therapeutic options in each single case.

The absence of randomisation and the small subgroups did not allow us a complete shift analysis. Finally, due to its retrospective nature, we were unable to assess some important aspects describing the impact of multimorbidity in the geriatric patient, such as previous cognitive function, previous functional and social status. Further studies assessing these aspects would be useful to clarify the impact of fibrinolysis and/or thrombectomy among multimorbid patients.

## Conclusion

This real-life experience shows the current treatment approach in elderly patients with AIS in a town of more than 100,000 inhabitants and underlines the need for reliable criteria for patient selection. Elderly subjects who did not receive interventional therapies showed worse functional outcomes and similar mortality to those treated with MT or IT. This finding seems to strengthen the indication for these approaches in older patients [15]. Larger studies are needed to obtain further information for improving acute treatment in the elderly also in light of the fact that newer and more specific devices are likely to improve MT outcomes, reduce operative time and lower mortality and complications.

## Data Availability

The datasets generated during and analysed during the current study are available from the corresponding author on reasonable request.
